# Remote EEG acquisition in Angelman syndrome using PANDABox-EEG

**DOI:** 10.1186/s11689-025-09611-x

**Published:** 2025-05-24

**Authors:** Kimberly Gálvez-Ortega, Roslyn Harold, Wei Siong Neo, Orlando S. Hoilett, Amanda M. Borosh, Alexa Friesen-Haarer, Stephanie Gombas, Dan Foti, Bridgette Kelleher

**Affiliations:** 1 Department of Psychological Sciences, College of Health and Human Sciences, Purdue University, 703 3rd Street, West Lafayette, IN 47906 USA; 2 College of Engineering, Purdue University, West Lafayette, IN USA; 3 College of Engineering and Applied Science, University of Cincinnati, Cincinnati, OH USA; 4 College of Education, Purdue University, West Lafayette, IN USA; 5 College of Education, Northern Illinois University, DeKalb, IL USA; 6 College of Arts and Sciences, Washington University, St. Louis, MO USA

**Keywords:** Telehealth, EEG, Angelman syndrome, Delta, Reliability, Assessment

## Abstract

**Objective:**

We describe the development and validation of PANDABox-EEG, a novel protocol for remote EEG assessment with no on-site technician, tailored for Angelman syndrome (AS). We argue that this protocol is reliable, valid, and widely acceptable for use in families affected by Angelman syndrome.

**Background:**

AS is a rare neurogenetic condition characterized by developmental delays, sleep problems, seizures, and a happy demeanor. People with AS are frequently monitored via EEG to inform clinical care, and EEG-measured delta activity has been proposed as a reliable biomarker to monitor treatment effectiveness. Traditional EEG assessments pose logistical and financial burdens for families due to the need to travel to a medical center to complete assessments. Telehealth methods, however, offer a pathway forward.

**Methods:**

PANDABox-EEG was developed through multidisciplinary collaboration with psychologists, psychophysiologists, engineers, and special-education scholars, incorporating caregiver feedback and user-centered design principles. It pairs PANDABox, a telehealth platform for biobehavioral assessment in rare disorders, with a dry electrode EEG system. Twenty-eight participants (7 AS, 7 siblings, 14 caregivers) completed three 5-min EEG sessions each over the course of a week. Caregivers were asked to provide feedback on acceptability of the design, and EEG data was quantified and assessed for metrics of reliability and validity.

**Results:**

PANDABox-EEG demonstrated high feasibility and acceptability, with 91% of caregivers reporting strong satisfaction assessment comfort. EEG data quality was promising, with high internal consistency (split-half reliability range for children with AS: *r* = .96-.98) and test–retest reliability for delta power among (test–retest reliability range for children with AS: *ρ* = .88-.96). Finally, we successfully detected the characteristic increased delta power in AS (effect size between AS and non-AS siblings: d = 1.56–2.85) and its association with age (effect size between non-AS siblings and caregivers: d = 2.19–2.72).

**Conclusion:**

PANDABox-EEG provides a feasible, cost-effective, and reliable method for remote EEG assessment in AS. Its high caregiver satisfaction and ability to capture relevant neurophysiological markers suggest potential for broader application. With further validation, PANDABox-EEG can enhance accessibility and inclusivity, benefiting clinical management and research in AS and other clinical populations in need of frequent EEG monitoring by eliminating the need to travel.

Angelman syndrome (AS) is a rare (1:20,000 [8]) neurogenetic syndrome caused by the loss of the maternal ubiquitin-protein ligase E3 A (UBE3 A) gene in the 15 th chromosome [21, 26]. As a result of the UBE3 A gene deficiency, individuals with AS experience a pronounced and severe clinical profile that includes motor impairments, intellectual disability, and sleep difficulties [2, 3, 7]. These features are most severely pronounced in patients with AS caused by deletion of *UBE3 A* gene, which occurs in approximately 70% of cases [5]. Seizures and sleep problems are nearly universally described in AS patients, with more than 80% experiencing seizures and 20–80% reporting sleep difficulties [38]. For this reason, seizures and sleep are consistently rated as highest priority for clinical trial targets by patient communities [33] due to their impact on patient and caregiver quality of life [37]. Given the importance of EEG in monitoring both sleep and seizure problems, patients with AS frequently engage in EEGs across their lives, including standard clinical care and research trials.

A robust literature has used EEG to characterize atypical brain activity in AS, dating back to the seminal publication introducing Angelman syndrome in 1965 [1]. Specifically, increased delta power has been shown to be a promising biomarker of AS, which is broadly associated with homeostatic processes [22] and may be observed to include “bouts” of spatially restricted delta and “notched” delta [29, 34, 35]. Within the AS community, elevated delta power effects are large and robust across genetic subtypes [13], present across the neocortex [29], change in response to treatment [25], predict cognitive function within AS [17, 28], are reliably detectable across short recording spans (< 8 min [30]), and persist regardless of sleep status and anti-seizure medication use [29]. Atypical delta power also follows a predictable developmental course, with greatest increases in early childhood [29, 30], and similar age-related decreases to non-AS patients [13]. Although other abnormal EEG findings are reported in AS patients [10], increased delta power collected during periods of wakefulness [24] produces the largest, most consistent, and most easily detectable difference across subgroups and ages [13, 24, 29], supporting its importance in both clinical management and trial-related outcome assessment.

The clinical trial landscape for AS is progressing rapidly, and EEG is a central component of most trials. The current status quo for EEG requires either patient travel to participate in EEG assessments at specialty clinical sites, or a partnership between trial investigators and specialty travel EEG companies that send technicians to collect data at patient homes. Nonetheless, these traditional methods have drawbacks and pose challenges in measuring EEG in AS patients as well as other patient communities affected by intellectual disabilities and sensorimotor impairments. Travel is highly burdensome to patients and potentially biases participation toward patients who are willing and able to travel, and away from patients without paid leave, childcare support, and historic mistrust of medical providers and settings [18]. In fact, research has suggested that individuals who are more likely to participate in physiological assessments, such as EEG, typically live within a 20-mile radius of the institution, leading to decreased EEG accessibility for those residing farther away [14]. Consequently, this is particularly isolating for low-income and geographically disbursed families who tend to be underrepresented in trials. With 21 dedicated specialty clinics for AS in the United States (www.angelman.org/clinics-directory-north-america), a minority of patients are able to easily access a local provider with specific expertise in AS. Travel EEG companies address some barriers by minimizing time and travel burden for families, however paying staff to travel is expensive, and most companies support trials for a variety of patient populations and may not have specific expertise working with AS patients. Therefore, alternative solutions are needed to reduce personal, financial, and geographic burden.

Remote technology offers a promising solution for improving the accessibility of EEG in AS and other patient groups. Indeed, a recent clinical trial found that AS caregivers preferred at-home EEG to in-clinic options [31], although the process for collecting EEG in that study required an on-site technician and was not fully remote. Despite the increasing availability of fully remote EEG systems [23], no published studies have successfully completed fully remote EEG with AS patients without a technician on-site. Reasons likely include the relatively new availability of fully remote systems, unclear reliability and validity of these systems in AS, and lack of patient-centered protocols necessary to support AS patients and their families to engage in these technologies. Stakeholder input is particularly important in AS due to the potential for patients to reject sensor-based assays due to sensitivities and curiosities that may be less common in other clinical samples. Indeed, others have reported unsuccessful use of wearable sensors in AS [15], paralleling well documented challenges of in-person EEG collection in a variety of neurodevelopmental groups [36]. Thus, adoption of a remote EEG protocol for AS trials will require substantial caregiver and community engagement to ensure technology is appropriate, acceptable, and feasible.

The present study introduces a novel, fully remote protocol for assessing EEG without a technician on-site. The protocol was developed for specific use in AS but is also anticipated to be applicable, with appropriate validation, to a variety of patient populations across the lifespan, with and without intellectual and developmental disabilities. Here, we report data from both AS patients and their unaffected family members to inform potential broader impact. To create this protocol, we expanded PANDABox (Parent Assisted Neurodevelopmental Assessment), a user-centered protocol for collecting biobehavioral data remotely in AS and other patient populations. PANDABox centers the patient’s caregiver as a key agent in the data collection process, reflecting caregivers’ expertise in their child’s behavioral needs and general interest in having a hands-on role in their child’s medical and clinical experiences [18]. Here, we describe the initial development and validation of the remote EEG extension of PANDABox (PANDABox-EEG) to address the following questions:

1. What is the feasibility of PANDABox-EEG in AS, as measured by observed ability of AS families to use PANDABox-EEG and by cost-savings analysis?
2. How do caregivers rate the ease of use, comfort, and perceived burden of PANDABox-EEG, as measured by post-assessment surveys?
3. Does PANDABox-EEG provide consistent and reliable estimates of delta activity within and between sessions, as measured by split-half reliability and test–retest reliability?
4. Does PANDABox-EEG provide a valid estimate of delta activity, as measured by its ability to capture the characteristic increased delta power in AS and expected age-related differences?

We hypothesized that PANDABox-EEG would be feasible, cost-saving, acceptable, and produce reliable data within sessions and across repeated assessments. We expected PANDABox-EEG to successfully recover increased delta power in AS patients relative to their similarly aged siblings and higher delta in power in children relative to adults.

## Methods

### Protocol selection and development

PANDABox-EEG was developed through a multidisciplinary partnership across clinical researchers, patients, caregivers, clinicians, and engineers. Here, we briefly describe the protocol development process prior to introducing the primary empirical validation study.

#### Initial patient community input

Across several interrelated projects, researchers engaged directly with patient communities to learn about past barriers to EEG data collection, including through communication at patient-focused conferences, an ongoing NIH-funded study of telehealth-based data collection in AS that uses wearable sensors [18] and focus groups on feasibility of wearable biosensors in AS and other patient populations [19]. Across engagements, stakeholders emphasized the need to minimize cords and facial fasteners, administer brief and simple tasks, allow ample time for patients and caregivers to acclimate to materials, and engage caregivers as active interest-holders in the assessment process. Altogether, these suggestions serve to reduce sensory overstimulation in people with AS, who are prone to sensory sensitivities; reducing this overstimulation would, we presumed, have the additional benefit of children with AS being more successful in sitting still (which is of great importance in EEG data collection, as movement artifacts are technically challenging or even impossible to correct for in post-processing). Thus, this input was centered in the development of the PANDABox-EEG protocol. For example, we developed an EEG Acclimation Kit to help patients comfortably engage with materials ahead of their sessions (described in “Materials” section).

#### Selection of user-centered design framework

The protocol was developed as a planned extension of PANDABox, a NIH-funded remote assessment system for collecting laboratory-grade data remotely in neurodevelopmental patient populations without an examiner on-site [18]. PANDABox specifies a standardized, user-centered approach to supporting caregivers to collect data from home and was specifically designed to accommodate the unique needs of rare disorder families. Components include clearly labeled materials that are sent to families in advance, visual prompts, live audio-visual coaching, and real-time monitoring of data fidelity by a trained examiner. PANDABox tasks are administered on a computer that is shipped to families and remotely managed by a live support coach who controls the pace of administration, displays instructions and prompts, and ensures data are collected with rigor and precision. Thus, caregivers are not tasked with managing technology and instead focus on supporting their child through the assessment. To maximize patient comfort, sessions are paused and restarted as needed. Given the nature of remote sessions, potential challenges, such as signal quality issues and distinct caregiver learning curves were carefully considered and monitored during assessments.

#### Selection and validation of EEG hardware system

The ANT Neuro eego sports™ amplifier with waveguard touch™ caps (Hengelo, Netherlands) was selected as the primary hardware system for PANDABox-EEG extension for several reasons. First, the system cap includes soft polymer electrodes rather than metal electrodes, maximizing patient comfort. Second, the cap does not require gel or water application, minimizing time for setup, caregiver facilitator demands, and messiness. Third, the system is compatible with TeamViewer, the teleconferencing software program used to communicate with caregivers during PANDABox assessments. Prior to selection, the research team conducted parallel assessments of the ANT Neuro system against gold-standard high-density EEG (actiCHamp system by Brain Products), both collected in a standardized laboratory setting. Resting-state EEG data was collected from one mother-infant dyad with typical development, as part of a separate longitudinal study. Results in this dyad indicated high concordance across systems (0.5–12 Hz at 0.5 Hz steps: time series correlation of *r* = 0.932 for mother [95% CI, 0.85–0.97] and 0.996 for infant [95% CI, 0.99–1]); while only a single dyad, these preliminary data consistent with the idea that the ANT Neuro system may accurately capture EEG power across the delta, theta, and alpha spectra.

### Study design

The present study employed a sibling- and caregiver-control, repeated-measure design to test performance of the PANDABox-EEG protocol in AS patients and their first-degree relatives. Sibling controls enabled us to test whether the protocol performed similarly across AS status (AS patients vs. siblings), and caregiver-controls enabled us to test whether the protocol performed adequately across the lifespan (AS patients and siblings vs. parents) in terms of data reliability and validity. Repeated observations enabled us to test the reliability of measurement across time.

### Participants and recruitment

Participants were recruited from existing internal participant registries and through conference presentations with two patient organizations, the Angelman Syndrome Foundation and Foundation for Angelman Syndrome Therapeutics. Interested individuals completed a phone screen to determine if the family met study eligibility. Probands with AS were required to be ages 6 months to 18 years, carry a caregiver-reported diagnosis of AS, and live in the continental United States. Families were required to additionally include a similarly aged sibling (< 3 years difference) and two in-home caregiving adults, all of whom were willing to participate in the study. Caregivers were all required to be legally able to consent for participation of both children. Participants were excluded for any serious illness, injury, or medical conditions that would interfere with study engagement and were not related to AS (e.g. severe visual impairment). All four members of the household were required to meet eligibility criteria for the family to participate. Eight families were enrolled in the study; one family withdrew due to scheduling conflicts prior to completing the first EEG assessment. This was the maximum sample size that could be collected due to the practical constraints of the pilot study. This sample size was adequate to yield preliminary data supporting the feasibility and acceptability of the protocol, as well as to achieve adequate statistical power (80%) for the confirmatory analysis of elevated delta in AS at large effect sizes (*d* > 1.50, one-tailed). Families received $10 per hour for participating in study activities.

### Procedure

Interested families contacted the research coordinator to conduct a brief screening interview to determine eligibility. Both caregivers were sent electronic consent forms and were required to separately complete demographic forms; verbal consent was also obtained at the beginning of the first family session. For siblings, physical copies of assent forms were sent to participant homes with the EEG equipment, reviewed in vivo with the project coach, and signed at the start of the first family session. Families were also asked to identify a primary caregiver who would answer study questionnaires and manage project communication. The primary caregiver conducted a brief pre-study survey to assess specific needs of their child with AS, including any sensorimotor constraints that could interfere with EEG assessment. Caregivers were offered an optional pre-assessment EEG Acclimation Kit to practice engaging with materials ahead of their scheduled session.

Once enrolled, families were mailed the PANDABox-EEG and EEG Acclimation Kits ahead of their scheduled session. At the beginning of the first family session, the examiner called the primary caregiver and supported them in setting up the testing equipment. Families were allowed to select the order of assessments across members but were encouraged to start with caregivers, which would enable the caregivers to gain hands-on exposure to the equipment prior to administering the session with their children. All families elected to complete all family member sessions on the same day. With the exception of AS patients, each family member signed a consent or assent form prior to their first EEG session; continued consent was confirmed prior to the start of each repeated session.

PANDABox sessions were facilitated by project coaches who were doctoral students with expertise in gold-standard, laboratory-based EEG acquisition. Each PANDABox-EEG administration followed the same sequence: (1) The project coach called the primary caregiver to confirm consent to continue with the project and help set up any kit components. (2) The project coach helped the primary caregiver identify a place in the home suitable for the EEG session. Suggestions included a table to spread out study materials, a nearby outlet to charge equipment, and relatively few distractions. (3) With the assistance of the project coach, the primary caregiver turned on the project tablets and TeamViewer software, which allowed the project coach to remotely assume control of the project tablets and administer tasks. (4) The project coach switched from phone to TeamViewer communication. (5) The project coach supported the caregiver in completing each step of the remote EEG sequence by providing verbal guidance, displaying stimuli and instructions, and monitoring data quality and administrating fidelity in real time. Each family member completed the EEG assessment sequence with support from the primary caregiver.

This sequence was repeated three times during the course of one week, producing 12 assessments per family (i.e., 4 family members assessed 3 times each). At the end of each session, caregivers were coached to properly store and charge equipment and were reminded of the time and date of the next session. Sessions were flexibly rescheduled as needed to maximize data collection quality and patient comfort, however, all testing sessions took place on different days (i.e., no two recordings for a single participant were recorded within a single day), and all recordings were conducted within the one-week span. Thus, the retest sessions could range between 1 and 5 days of each other for any two sessions, and between 2 and 7 days of each other for the first and last session.

After data collection was complete, families were instructed to disassemble study components and place the PANDABox-EEG and acclimation kits outside of their home for a pre-scheduled, prepaid pickup by a mail delivery carrier. Families were also sent follow-up surveys on their experiences during their PANDABox-EEG sessions.

### Materials

#### Caregiver questionnaires

Caregivers completed separate questionnaires before and after their EEG sessions. Pre-assessment questionnaires assessed demographic information, child behavior (e.g., “How similar do you think their behavior would look at a research lab with a research assistant?”), technology comfort (e.g., “How would you rate your comfort with Skype, Facetime, or other video chat technology?), access to resources/materials (“Do you have access to wireless internet (wifi) in your home?”), child sensory sensitivities (e.g., “How well is your child WITH Angelman syndrome able to wear a hat? How well do you think your child WITH Angelman syndrome will be able to have an EEG cap for 15 min?”) and potential support needs (e.g., “Do you think it would be helpful to your family if either of your children or you could practice wearing an EEG cap prior to recording?”). Post-assessment questionnaires were distributed anonymously and measured perceived satisfaction with the PANDABox-EEG protocol.

#### PANDABox-EEG kit

**Fig. 1:**
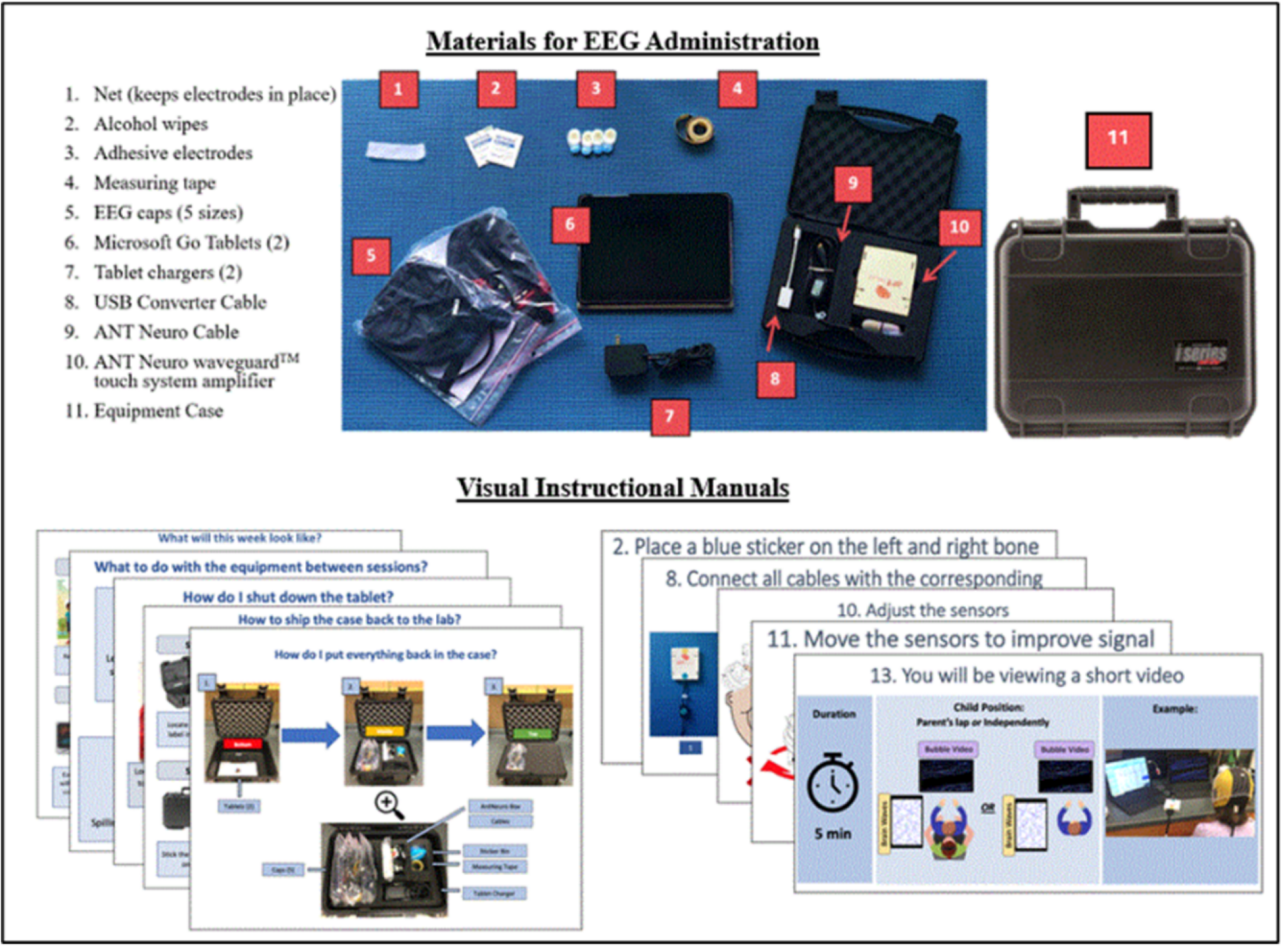
Overview of the PANDABox-EEG Kit components, including the equipment shipped to families (top) and visual instructional materials to facilitate the EEG sessions (bottom)

Kit components are depicted in Fig. 1. The remote assessment kit included the following components: (1) visual instructional manuals that described the timeline of visits and general instructions; (2) a Microsoft Go tablet for EEG data collection and videoconferencing, (3) a Microsoft Go tablet for stimulus display, (4) the ANT Neuro eego sports™ system with waveguard touch™ caps in a range of child and adult sizes, (4) labeled materials for preparing the EEG (e.g., measuring tape, mastoid electrode stickers, alcohol wipes, etc.), and (5) a return shipping label. Where possible, equipment was pre-connected with labeled cords to minimize family burden. Computers were equipped with TeamViewer software (TeamViewer GmbH, Göppingen, Germany), ANT Neuro eego software, and Microsoft Office. Tablet desktops were cleared of irrelevant applications and included prominent shortcuts to key software, a PowerPoint instruction deck, and a link to digital consent forms and surveys. Equipment was transported in industrial-grade foam custom-shaped to fit project components, and a sturdy, locked equipment box. Each kit cost $18,434 (2021 price) including EEG equipment, ancillary materials, and shipping materials.

#### ANT neuro eego sports™ system with waveguard touch™ caps

This system uses passive, dry, Ag/AgCl electrodes. Each electrode consists of 30 pins of 1 mm diameter that are designed to pass through the hair and maximize scalp contact. Pin height varies across electrode based on scalp position in order to minimize pressure points and optimize fit. The cap is a polyester fiber mesh, and all electrodes are sewn into fixed positions within the cap. The current study used an 8-electrode montage, corresponding to scalp locations of Fpz, Fz, F3, F4, Cz, C3, C4, and Pz. Two additional electrodes were placed at the mastoids using disposable sensors embedded within an adhesive and designed for one-time usage. The EEG was sampled at 500 Hz. During recording, the online ground was the left mastoid electrode and the reference was the right mastoid electrode; no filter was applied during recording.

#### EEG acclimation kit

**Fig. 2:**
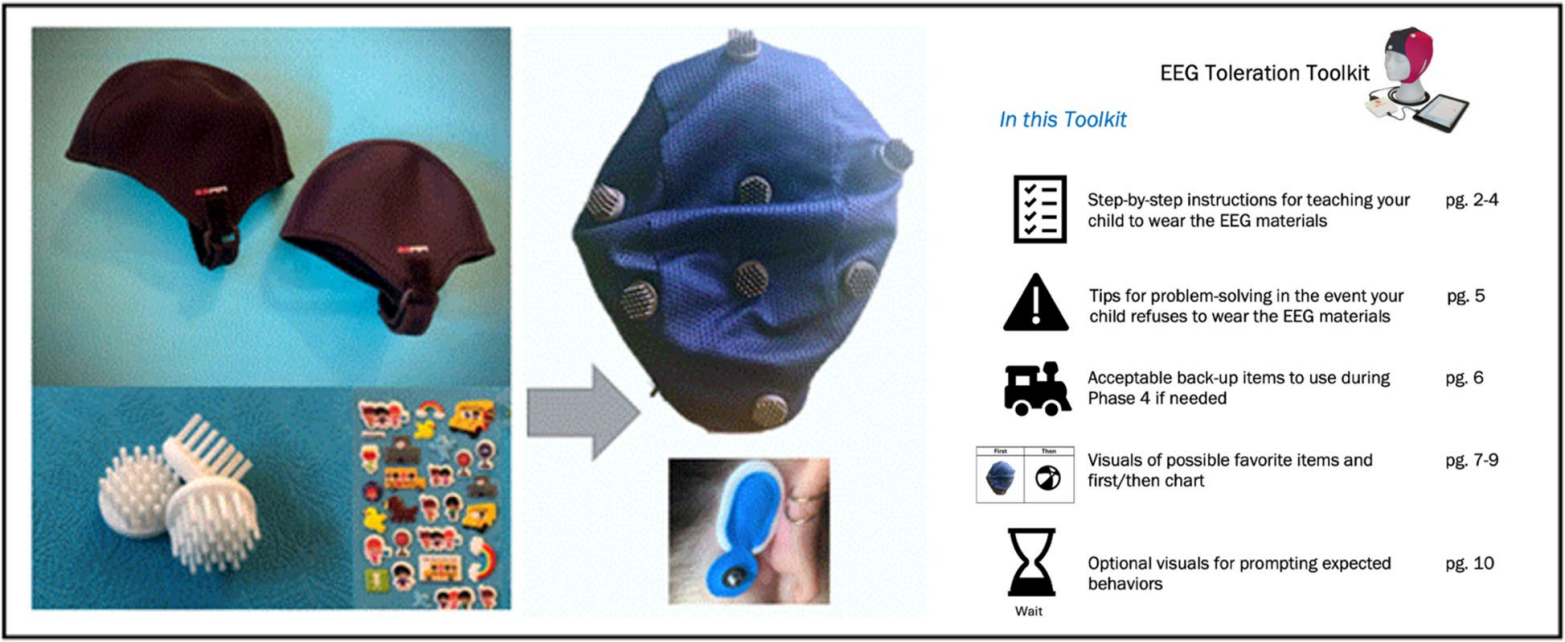
Components of the EEG acclimation kit which was delivered to families in advance to facilitate physical comfort with the dry-electrode EEG caps. The acclimation kit included neoprene caps with 3D-printed electrodes and sticker sheets (left) designed to mimic the tactile sensation of the dry-electrode EEG caps and mastoid sticker electrodes (middle). Also included was a printed guide for caregivers to use the acclimation kit with their child (right)

Acclimation kits were developed by experts in clinical management of challenging behaviors (AB), biomedical engineering (OH), and EEG acquisition (RH/KGO), based on initial input from patient community stakeholders (Fig. 2). Realistic practice caps were created by attaching 3D-printed faux electrodes—which mimicked the sensation of real electrodes—to neoprene caps that felt similar to the ANT Neuro waveguard^TM^touch system. Separate sheets of kid-friendly stickers were included in the acclimation kit to mimic the sensation of the two adhesive electrodes placed at the mastoids. Each family received two neoprene caps in different child sizes with several sheets of stickers, as well as the following materials: (1) step-by-step instructions for teaching a person with AS to wear the cap, (2) tips for problem-solving in the event the person with AS has difficulty wearing the cap (3) examples of backup items or activities to help children transition between tasks, (4) a “first/then” behavioral schedule that the families could use to present and select rewards for EEG participation, and (5) visuals cards to prompt desired behaviors involved in EEG (e.g. hands on lap, sitting, waiting).

#### Host laboratory

Project coaches teleconferenced from Purdue University. The laboratory included two Dell OptiPlex 5050 computers equipped with TeamViewer software, a webcam, and a landline phone. The laboratory was in a quiet and secure location designed for clinical assessments.

#### Data acquisition and task

**Fig. 3:**
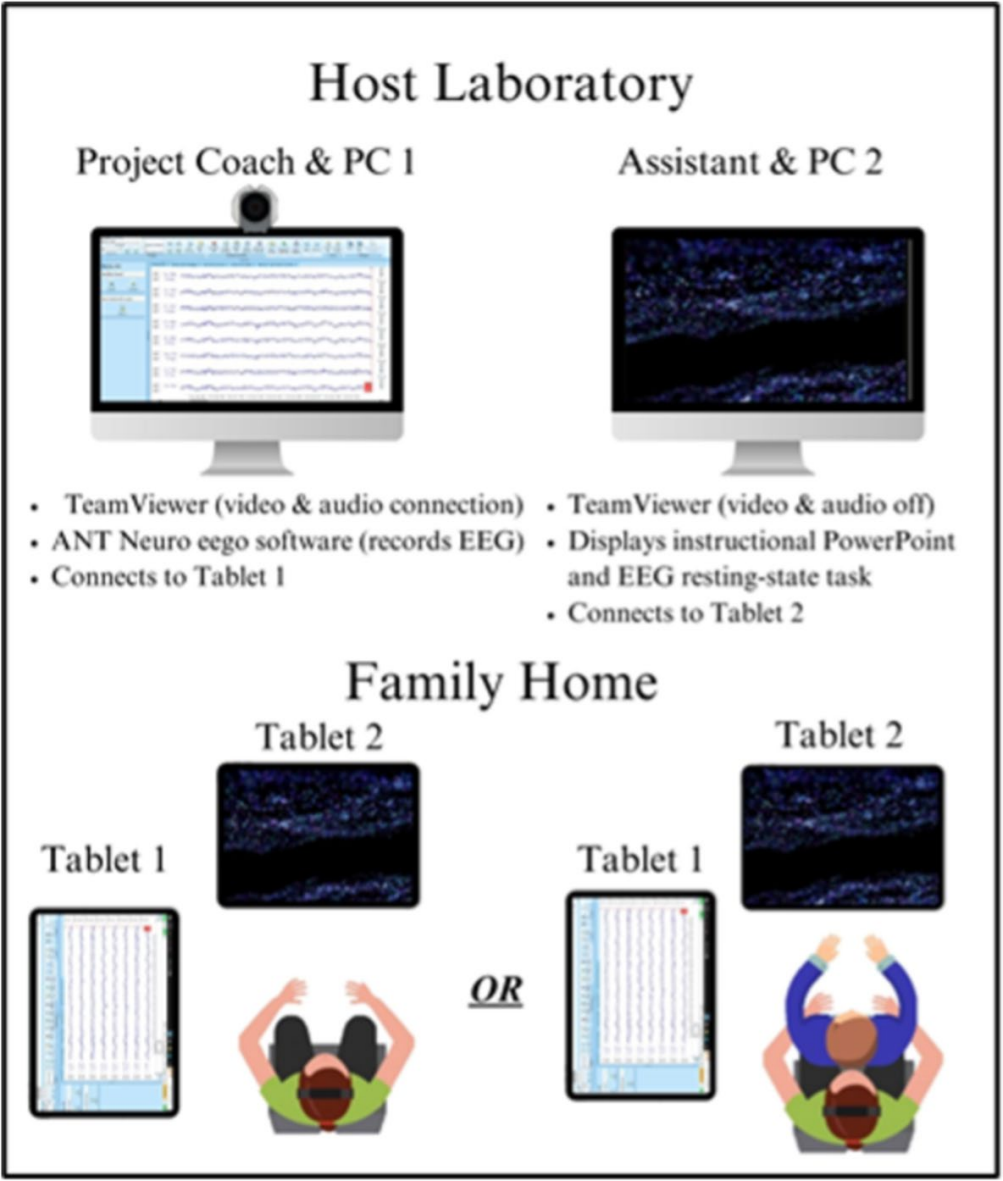
Equipment and personnel configurations within the laboratory (top) and at families’ homes (bottom) during EEG acquisition

Configurations of equipment and personnel during EEG acquisition are depicted in Fig. 3. First, participants were coached to apply the EEG cap. Next, the project coach remotely supported the primary caregiver to adjust cap placement until electrode impedances were satisfactory. They then completed the resting state task with their eyes open. Data quality via visual inspection was monitored in real time by the project coach—one of two advanced doctoral students with expertise in EEG data acquisition—to ensure appropriate data quality.

#### Resting state task

Participants completed a 5-min, eyes-open resting state task while the continuous EEG was recorded. Though it is typical to use a fixation cross for resting scans in adult populations, a silent video of floating geometric fractals was selected to be developmentally appropriate for AS patients and siblings [16]. Participants were permitted to sit in their caregiver’s lap or independently. Participants were monitored for signs of sleep or overt seizures during the task.

#### Signal processing

EEG data were processed offline, after equipment was returned to the laboratory. Visual inspection of individual channels within each recording session was conducted by two expert raters (RH, DF). Channels judged to be of poor quality for a majority of the scan were rejected for that scan prior to data reduction. This included rejecting electrodes which did not achieve connection with the scalp, or those which had persistent physiological artifacts throughout. Every rating achieved consensus prior to rejection or inclusion. Electrodes were not interpolated due to the cap only including 8 electrodes.

Next, EEG data were processed consistent with prior EEG research in AS [29]. First, data were filtered using a band pass filter between 0.1 Hz and 100 Hz with an 8 th order roll off, and a notch filter was applied at 60 Hz to account for electrical noise. Data were then segmented into 1 s intervals with 50% overlap. Next, physiological artifacts within individual segments were identified automatically based on criteria of (a) a voltage jump of > 50 µV across consecutive samples, (b) a change of < 0.5 µV within a segment, or (c) a change of > 150 µV within a segment. Next, a Fast Fourier Transform was applied with a resolution of 0.5 Hz. This transform was set to output power (µV^2^) with only simple output (i.e., spectral line values; phase information was not considered), with a 50% symmetric hamming window. Artifact-free segments were averaged separately for each electrode, session, and participant. Delta power was quantified as the average between 2 and 4 Hz.

### Cost–benefit analysis

Cost–benefit analysis procedures were modeled after a prior report from our group [18] to estimate the travel and equipment costs that vary across in-person and remote studies. For remote assessments, the number of PANDABox-EEG kits needed were estimated using an 11-day shipping and repackaging window; 1 kit was estimated to be needed for 1–2 family assessments/month, and 2 kits for 3–5 family assessments/month. Costs of patient travel were estimated as the average round-trip flight cost to our local airport ($385.96 × 4 passengers, United States Department of Transportation, 2024), and lost wages as the average daily wage in the United States ($163.29, United States of Labor, 2024), multiplied by 1.6 to account for employer loss of productivity. Relative costs of space and clinical staff are not considered in the analyses, consistent with prior work. Travel costs are estimated using 4 nights and 5 days of travel. Cost–benefit analyses did not consider staffing and space charges, which are anticipated to be higher for in-person assessment models.

### Analytic plan

Pre-assessment comfort, feasibility, and acceptability outcomes were descriptive in nature; we pre-set a criterion of 80% feasibility and acceptability as successful for the purposes of our analysis. Cost-saving analysis projections were also descriptive and generated for a 12-month period during which between 1–5 families were projected to be assessed per month. Internal consistency analyses of EEG data were conducted using the Spearman-Brown prophecy formula for split-half reliability, calculated from averages of even and odd segments, following prior literature in adult and pediatric populations [27, 32]. Test–retest reliability of delta power was calculated using Spearman rank correlation— due to its relative robustness against small sample sizes [9]—between sessions in the same group (AS child, sibling, caregivers). Validity of expected differences in delta power were assessed descriptively, and correlations were used to assess the degree to which delta power changed across age within the AS group and their siblings.

### Ethical statement

The project was approved by the Purdue University Institutional Review Board (no. IRB- 2021–53). All adult participants provided written consent, and pediatric participants ages > 8 without intellectual disabilities provided written assent.

## Results

### Participant characteristics

Participants included 28 total individuals from 7 families: 7 children with AS, 7 similarly-aged siblings, and 14 caregivers. Demographic information is included in Table 1. Caregivers reported a median income of $60,000 and lived an average of 857 miles from the laboratory (range 39—2045).

**Table 1:** Participant characteristics

	Angelman Syndrome		Siblings		Caregivers	
	N	%	N	%	N	%
Race/Ethnicity						
* Hispanic/Latine*	1	14.3	1	14.3	1	7.1
* Native Hawaiian*	0	0	0	0	1	7.1
* White*	6	85.7	6	85.7	12	85.7
Sex						
* Females*	2	28.6	2	28.6	7	50
* Males*	5	71.4	5	71.4	7	50
	*M (SD)*	Range	*M (SD)*	Range	*M (SD)*	Range
Age, chronological	6.29 (4.19)	2–15	6.86 (5.21)	2–17	37.07 (5.15)	26–46
Age, developmental	2.5 (0.97)	0.75–3.5	–	–	–	–

Child developmental age based on caregiver report

### Pre-assessment technology and EEG comfort

Pre-assessment surveys characterized participants’ prior experiences with technology and EEG. Caregivers generally had prior experience with EEG, either directly (*n* = 2) or through observation (*n* = 10). A majority of caregivers thought it would be helpful for their family to practice wearing an EEG cap (yes = 8, no = 2, maybe = 1, missing = 3) or stickers behind their ears (yes = 8, no = 2, maybe = 1, missing = 3) prior to the session; when offered the EEG acclimation kit to prepare for session, all families accepted.

Primary caregivers were also prompted to rate their comfort with technology, as well as the likely tolerability of EEG assessments for themselves, their child with AS, and their child without AS (Table 2). Caregivers generally reported good to excellent comfort with video chat technology, tablet devices, video cameras, and learning new software. Caregivers generally reported good to excellent anticipated tolerability of the EEG assessment process for themselves and for their child without AS. As expected, caregivers rated the anticipated tolerability of the EEG process as lower for their child with AS, with the majority reporting “Poor” or “Fair” anticipated tolerance wearing items like hats or headphones.

**Table 2:** Pre-assessment survey on comfort with technology and EEG assessment

	Poor	Fair	Good	Excellent	Missing
How comfortable are you with:					
* Video chat technology*	0	1	2	4	0
* Tablet devices*	0	0	2	4	1
* Video camera use*	0	0	3	3	1
* Learning new software*	0	0	2	4	1
How well do you think you will:					
* Place an EEG cap on yourself*	0	1	1	9	3
* Place an EEG cap on your child with AS*	0	1	3	6	4
* Place an EEG cap on your child without AS*	0	0	4	6	4
* Stare at fixation cross for 5 min*	0	1	1	8	4
How well will your child with AS:					
* Wear a hat*	1	4	2	0	0
* Wear headphones*	1	4	2	0	0
* Have their hair brushed or combed*	0	2	1	4	0
* Have an EEG cap on for 15 min*	0	3	2	2	0
* Have stickers placed behind their ears*	1	1	2	3	0
* Sit still with an EEG cap for 5 min*	1	2	2	2	0
* Watch a video for 5 min*	0	2	5	0	0
How well will your child without AS:					
* Wear a hat*	0	0	1	6	0
* Wear headphones*	0	0	2	5	0
* Have their hair brushed or combed*	0	0	0	7	0
* Have an EEG cap on for 15 min*	0	0	2	5	0
* Have stickers placed behind their ears*	0	0	2	5	0
* Sit still with an EEG cap for 5 min*	0	0	2	5	0
* Watch a video for 5 min*	0	0	1	6	0

Numbers indicate the frequency of endorsement. All items were administered to the primary caregiver, and a subset of items were also administered to the secondary caregiver

### Feasibility

#### Session completion

Of the 84 planned assessments (12 assessments per family for 7 families), all but 1 caregiver assessment were completed as planned, with 3 reschedules or adjustments to timing due to family needs. Family assessments did not extend beyond the one-week period. All participants completed the resting state task for each session, with no observed sleep, drowsiness, or overt seizures during the brief recording period.

#### Equipment damage/loss

One acclimation kit was lost during transit from the lab to the family. All PANDABox-EEG kits were successfully returned to the lab without damage.

#### Cost–benefit analysis

Estimated cost-savings of EEG assessment conducted using the PANDABox-EEG kit versus traditional, laboratory-based EEG assessment are presented in Table 3. Across one year of assessments, it was estimated that remote EEG would save between $41,515 (1 family/month; 12 total) and $222,427 (5 families/month; 60 total), without taking into account lost wages (estimated at $2,613 for in-person versus $522 remote assessments) and additional staffing and facility costs associated with in-person assessments and clinics.

**Table 3:** Cost–benefit analysis for remote versus in-person EEG

	Laboratory	Remote	Cost Savings
Cost by project element			
Fixed Monthly Costs			
* System Costs (1–2 Assessments/Month)*	$18,434	$21,914	-$3,480
* System Costs (3–5 Assessments/Month)*	$18,434	$43,828	-$25,384
* HIPAA compliant telehealth software (monthly)*	$0	$233	-$233
Family-Specific Costs			
* Flights ($386 each)*	$1,544	$0	+ $1,544
* Baggage ($80)*	$80	$0	+ $80
* Airport parking ($23/day)*	$115	$0	+ $115
* Airport transportation ($150 per trip)*	$150	$0	+ $150
* Lodging ($135/day)*	$675	$0	+ $675
* Meals ($64/day each)*	$1,280	$0	+ $1,280
* Remuneration ($90/family)*	$90	$90	$0
* Remote Kit Shipping ($75)*	$0	$75	-$75
* Caregiver lost wages ($29/hour each)*	$2,613	$522	+ $2,091
Projected researcher cost for 12 months of assessments, 1–5 families per month (12–60 families total)			
1 Family	$65,642	$24,127	+ $41,515
2 Families	$112,850	$26,107	+ $86,743
3 Families	$160,058	$28,087	+ $131,971
4 Families	$207,266	$30,067	+ $177,199
5 Families	$254,474	$32,047	+ $222,427

^*^Due to shipping times, it is estimated that 1 remote EEG kit can be used for up to 2.5 assessments per month. Although both in-person and remote assessments use the same EEG system, two additional computers are required for each remote administration. Projected costs are isolated to those directly incurred by the research team and do not include lost wages; Projected Cost = (fixed monthly cost) + (n families/month)*[(family-specific costs) – (lost wages)]

### Acceptability

Of the 7 families that completed the EEG assessments, the primary caregiver from 6 families completed an anonymous post-assessment survey of satisfaction with study procedures (Table 4). Caregivers generally reported a high level of acceptability for the EEG assessment sessions, with 91% of item responses rated “Excellent.” Applying the EEG cap was rated as “Poor” for one child and “Fair” for one caregiver.

**Table 4:** Post-assessment ratings of study procedures

Satisfaction with EEG assessments	Poor	Fair	Good		Excellent	Missing
Voice quality of assessment materials	0	0	0		6	0
Visual quality of assessment materials	0	0	0		6	0
Ease of technology use	0	0	0		6	0
Ease of getting the cap on your child	1	0	2		3	0
Ease of getting the cap connected to the tablet	0	0	0		6	0
Ease of adjusting the electrodes on your child	0	0	2		4	0
Ease of getting the cap on yourself	0	1	0		5	0
Ease of adjusting the electrodes on yourself	0	0	2		4	0
Length of time participating	0	0	0		6	0
Personal comfort of the assessment to your child	0	0	0		6	0
Personal comfort of the assessment to you	0	0	0		6	0
Overall experience with the assessment	0	0	0		6	0
Clarity of instructions/directions	0	0	0		6	0
Amount of support from our team	0	0	0		5	1
Ability of our team to answer your questions	0	0	0		6	0
Sensitivity and friendliness of our team	0	0	0		6	0
Privacy of the assessment	0	0	0		6	0
Ability to capture your child's abilities	0	0	1		5	0
Ability to capture your child's typical behavior	0	0	1		5	0
Comparison of remote and past in-person EEG experiences for child with AS	About the Same	Slightly Better	Moderately Better		Much better	Missing
Time spent setting up the EEG	0	1	1		3	1
Time spent doing (recording) the EEG	0	1	0		4	1
The comfort of the EEG	0	1	1		3	1
Ability to capture your child's abilities	1	0	1		3	1

Survey responses were collected anonymously from families after completing all other study procedures

When asked how the remote EEG experience for the current study compared to past experiences with in-person EEG assessments for their child with AS, all caregivers rated remote assessment as more favorable regarding the time spent setting up, time spent recording, and the comfort of the EEG for their child with AS.

### Reliability

The psychometric properties of delta power are presented in Table 5. Internal consistency of delta power was excellent for all three EEG assessment sessions among children with AS, siblings, and caregivers.

**Table 5:** Psychometric properties of delta power assessed remotely

Internal Consistency Within Sessions						
	Angelman Syndrome		Non-AS Siblings		Caregivers	
	N	Reliability	N	Reliability	N	Reliability
Session 1	7	.980	7	.998	13	.997
Session 2	6	.966	7	.997	12	.993
Session 3	7	.982	7	.998	14	.996
Test–retest Reliability Across Sessions						
	Angelman Syndrome		Non-AS Siblings		Caregivers	
	N	Spearman’s *ρ*	N	Spearman’s *ρ*	N	Spearman’s *ρ*
Session 1–2	6	.943	6	.257	12	.671
Session 2–3	6	.886	6	.314	12	.203
Session 1–3	7	.964	7	.750	13	.242

Internal consistency values are split-half reliability, calculated using the Spearman-Brown Prophecy formula for odd and even segments

Test–retest reliability of delta power varied in strength between sessions and groups. Delta power was highly stable among children with AS across all three assessments. Stability estimates were lower overall among siblings and caregivers, indicating greater fluctuations in delta power across assessments. Among siblings, high stability was observed between sessions 1–3. Among caregivers, high stability was observed between sessions 1–2.

### Validity

**Fig. 4:**
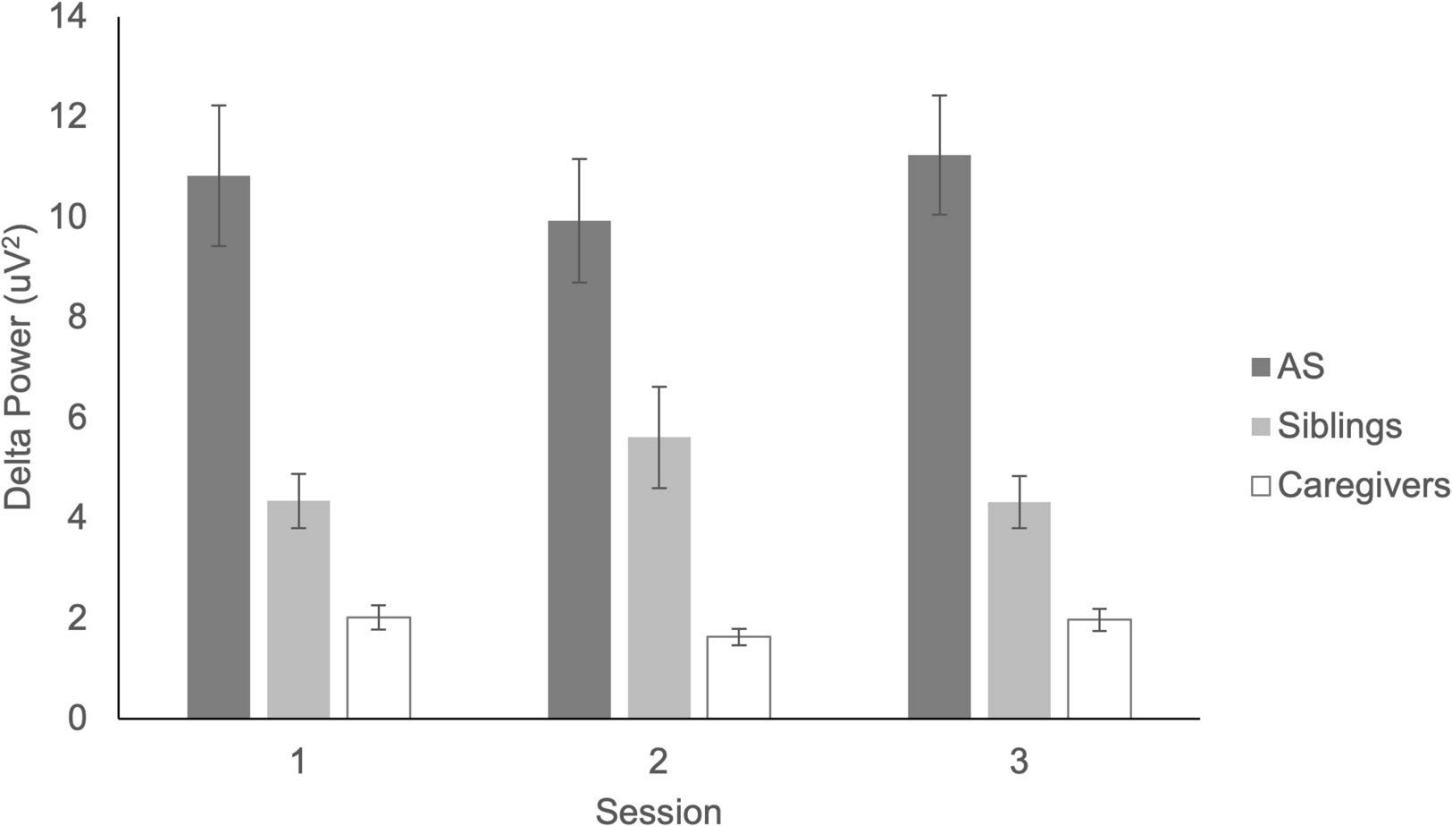
Delta and power across recording sessions, presented separately for children with Angelman Syndrome (AS), their unaffected siblings, and caregivers. Error bars depict the standard error of the mean

Descriptive comparisons of delta indicated large size differences between AS and sibling controls, and between sibling controls and adult caregiver controls (Table 6 and Fig. 4). As expected, delta power was increased among children with AS compared to their unaffected siblings, with large effect sizes.

**Table 6:** Effect sizes of group differences in EEG power

	Angelman Syndrome vs. Non-AS Siblings	Non-AS Siblings vs. Caregivers
Session 1	2.303	2.119
Session 2	1.566	2.728
Session 3	2.852	2.266

Values are Cohen’s *d*

**Fig. 5:**
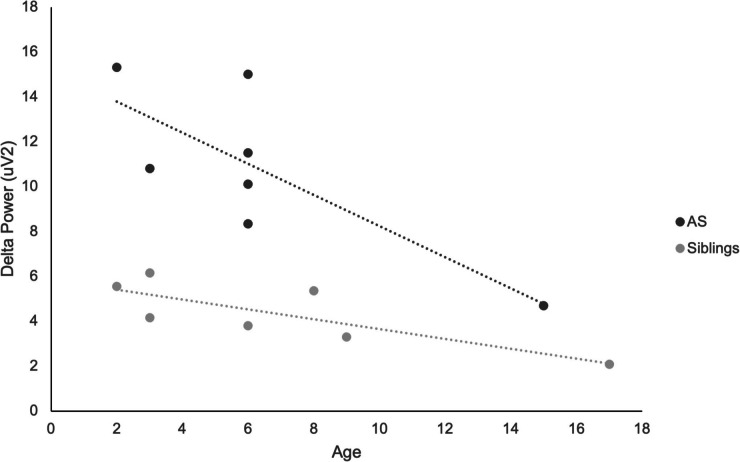
Scatterplot depicting the association between delta power at the first EEG session and chronological age, among children with Angelman Syndrome and their unaffected siblings

Nonparametric correlations indicated that delta power decreased with chronological age among AS children (Spearman’s rho: − 0.709 at Session 1, − 0.820 at Session 2, and − 0.571 at Session 3; also see Fig. 5) and their siblings (− 0.811 at Session 1, − 0.348 at Session 2, and − 0.631 at Session 3), confirming that the phenotypic characteristic of increased delta power was most prominent in early childhood.

## Discussion

The present study introduces and validates the PANDABox-EEG protocol, a fully remote, technician-free system for acquiring EEG data in individuals with Angelman syndrome (AS). Increased delta power, collected via EEG, is a promising biomarker for AS given its robust and well-characterized developmental profile and sensitivity to both developmental and treatment related changes. However, collecting EEG from AS patients is cumbersome and expensive, posing a barrier to patients’ day-to-day clinical care, as well as the development of representative, high-powered clinical trials. Here, we present PANDABox-EEG, a novel protocol for collecting EEG from AS patients remotely, without a technician on-site. Outcomes suggest that PANDABox-EEG is feasible, cost-saving, and acceptable to caregiver stakeholders. Data collected using this system are reliable for AS patients within- and across sessions and recover the expected AS EEG phenotype. Here, we contextualize these findings with past research and discuss how PANDABox-EEG may potentially facilitate more streamlined, affordable, and inclusive clinical care and trials for patients with AS.

### Feasibility and acceptability

We observed high completion rates and positive feedback from caregivers, underscoring the feasibility and acceptability of the PANDABox-EEG protocol. Caregivers reported strong satisfaction with the ease of technology use, comfort of the assessment, and overall experience. These findings suggest that user-centered design principles used in this project were effective in accommodating the unique needs of AS patients. This work complements a variety of past research studies noting the utility of various acclimation protocols in facilitating EEG with neurodiverse populations [6, 11]. Here, we demonstrated that such principles could be implemented for remote-only administration. Points of emphasis for the design of the current protocol were an EEG acclimation kit designed to mimic the specific tactile experience of the electrodes, the incorporation of customized visual aids for caregivers, and streamlining the hardware and software to be used during EEG data acquisition.

Cost–benefit analyses revealed significant cost savings associated with the remote PANDABox-EEG protocol compared to projected costs of traditional in-person assessments. Whereas the current protocol incurred additional expenses for dedicated remote software and support computers, these were more than offset by the costs that would typically be incurred for participant travel for in-person EEG assessments. These results suggest that by eliminating travel and on-site technician requirements, the protocol may reduce both direct costs and indirect burdens on families, such as lost wages and travel-related stress. These savings may be particularly meaningful for families from low-income or geographically isolated backgrounds, who are often underrepresented in clinical research due, in part, to logistical and financial barriers.

### Reliability and validity

In addition to demonstrating high feasibility and acceptability, promising findings were also observed in terms of reliable EEG data and the identification of clinical and developmental characteristics of EEG activity. For instance, the PANDABox-EEG protocol produced high reliability of delta power scores, as shown through estimates of excellent internal consistency for all three EEG assessment sessions and for all groups, as well as robust test–retest reliability of delta power for patients with AS. These parameters suggest that PANDABox-EEG aligns with field standards for research-grade data, including requirements that biomarker assays for clinical trials produce adequate psychometric characteristics (Webb et al., 2023). Whereas delta scores were highly stables for patients with AS across the three EEG assessments, they were lower overall for unaffected siblings and caregivers, indicating more variability in these groups across sessions. This variability can likely be attributed to state differences across the three assessments, such as wakefulness. Because this was a preliminary study focused primarily on the feasibility and acceptability of the protocol, EEG sessions were arranged primarily around the participating families’ scheduled. Future studies could reduce the influence of state differences by conducting EEG sessions at fixed times and by taking into account potential confounding effects of sleep and medication status. The values for temporal stability presented here should be interpreted with caution due to the relatively small sample size for this initial study.

We also observed several promising indicators of validity of the EEG assessments conducted as part of the current study. First, the protocol successfully detected the characteristic increased delta power in AS patients, aligning with previous research [25, 28, 30]. Elevated delta was most prominent among younger children with AS and decreased with age, as has been reported previously [29]. Further, the protocol also recovered expected developmental differences between children and adults [11], as indicated by elevated delta power among the unaffected siblings compared to their caregivers. Altogether, this pattern of effects validates PANDABox-EEG’s capability to identify key biomarkers of the syndrome and suggests that it appears to be identifying the expected clinical and developmental features of EEG activity within the delta band.

### Next steps for development

Despite the success we observed in using the PANDABox-EEG protocol in the present study, it is notable that our small sample predominantly included white and affluent families who were connected to our study through patient organizations. It is well established that demographically diverse families are under-represented in rare disorder research [12]; thus, it will be important to probe the utility and uptake of PANDABox-EEG in other demographic subgroups prior to widespread use, including patients that may not be affiliated with patient organizations and registries. It will also be important to ensure the physical equipment we use is acceptable to families from diverse backgrounds. For example, EEG assessments among Black individuals are affected by differences in the physical qualities of Black hair, the cultural significance of Black hair, and the broader context of anti-Black racism [4]. As a step toward ensuring PANDABox-EEG will be appropriate for users from diverse backgrounds, we are currently translating PANDABox-EEG to Spanish and testing its acceptability in Spanish-speaking populations, paralleling recent work translating the primary PANDABox battery to Spanish [20].

It will also be important to determine the limits of this system in capturing EEG-related biomarkers in AS. Although PANDABox-EEG was reliable and valid for assessing delta power, a variety of other biomarkers are relevant to the AS phenotype. For example, AS patients also demonstrate atypical gamma coherence and sleep spindles during periods of sleep [10] and abnormal clinical EEG features such as intermittent rhythmic delta and theta waves, interictal epileptiform discharges, and posterior rhythm slowing [35]. A next step will be to validate PANDABox-EEG for collection of broader EEG features relevant to AS in a larger, more demographically diverse cohort, which will permit understanding of the technological limits of this battery in supporting the needs of the AS community. Future iterations of the protocol may also consider modified equipment to capture additional EEG features (e.g., a modified electrode montage to capture seizure-related activity at occipital regions), as well as a modified behavioral protocol to capture EEG activity over a longer span of time.

### Conclusion

The PANDABox-EEG protocol represents a significant advancement in the field of remote neurophysiological assessment by providing a feasible, reliable, and cost-effective method for collecting EEG data without the need for specialized technicians or travel. Increasing access to reliable and acceptable EEG assessments for communities experiencing personal, financial, and geographic burden has the potential to transform clinical and research practices for AS and other neurodevelopmental disorders. Specifically, this sets a path for greater participation in EEG assessments, more representative samples, more informed patient care, and potential treatment advancements. While the PANDABox-EEG protocol does not come without its limitations, the high levels of caregiver satisfaction and the protocol's ability to capture key EEG biomarkers further support its utility and applicability. Future validation studies may wish to evaluate its use in assessing other key clinical biomarkers in a larger and more demographically diverse cohort. In summary, the PANDABox-EEG protocol offers a practical and effective solution for remote EEG data collection, providing an inclusive and accessible option that could significantly benefit clinical and research efforts in AS and beyond.

## Data Availability

The deidentified datasets supporting the conclusions of this article will be made available upon request.
